# Challenges and Strategies for Mainstreaming Neglected Tropical Diseases Campaign Interventions in Ethiopia

**DOI:** 10.4269/ajtmh.24-0261

**Published:** 2024-11-26

**Authors:** Awraris Hailu, Teshome Gebre, Fikre Seife, Wendemagegn Enbiale, Adugna Tamiru, Tsegaye Yohanes, Amsayaw Yohannes, Melese Kitu, Behailu Merdekios, Biruck Kebede, Fikreab Kebede, Kebede Deribe, Matthew J. Burton, Esmael Habtamu

**Affiliations:** ^1^ Eyu-Ethiopia: Eye Health Research, Training & Service Centre, Bahir Dar, Ethiopia;; ^2^ School of Public Health, Asrat Woldeyes Health Sciences College, Debre Berhan University, Debre Berhan, Ethiopia;; ^3^ The Task Force for Global Health, Addis Ababa, Ethiopia;; ^4^ National NTDs, Program, Disease Prevention and Control Directorate, Ethiopia Ministry of Health, Addis Ababa, Ethiopia;; ^5^ College of Medicine and Health Sciences, Bahir Dar University, Bahir Dar, Ethiopia;; ^6^ Collaborative Research and Training Center for NTDs (CRTC-NTDs), Arba Minch University, Arba Minch, Ethiopia;; ^7^ Research and Community Engagement, Arba Minch University, Arba Minch, Ethiopia;; ^8^ RTI International, Addis Ababa, Ethiopia;; ^9^ Children’s Investment Fund Foundation, Addis Ababa, Ethiopia;; ^10^ International Centre for Eye Health, Clinical Research Department, Faculty of Infectious and Tropical Diseases, London School of Hygiene & Tropical Medicine, London, United Kingdom;; ^11^ National Institute for Health Research Biomedical Research Centre for Ophthalmology at Moorfields Eye Hospital NHS Foundation Trust and UCL Institute of Ophthalmology, London, United Kingdom

## Abstract

Mainstreaming neglected tropical diseases (NTDs) interventions in national health systems is one of the key strategies emphasized in the WHO Roadmap for NTDs. However, there is limited evidence on implementing the proposed mainstreaming approaches effectively. We used a participatory ranking methodology in Ethiopia, using consultative workshops with purposively selected stakeholders, including NTDs program leaders from the government and partners, primary health care (PHC) workers, and community leaders and volunteers. Our aim was to identify, rank, and contextualize mainstreaming challenges and strategies, which were then synthesized using the Primary Health Care Performance Initiative framework. Thirty-three stakeholders at the national, regional, district, PHC, and community levels participated in two consultative workshops conducted in the Adama and Shashemene towns in Ethiopia. The stakeholders identified 73 mainstreaming challenges related to service delivery (32 [43.8%]), inputs (22 [30.1%]), systems (18 [24.7%]), and outputs (1 [1.4%]). The top three most frequently cited and ranked challenges were poor data recording and reporting, poor drug management and logistics, and weak supportive supervision and monitoring. Among the 185 strategies identified to address these challenges, the three most frequently cited were establishing a strong, supportive supervision and monitoring system, continuous on-the-job training to build workforce competence, and performance-based motivation. Multifaceted NTDs campaign intervention mainstreaming challenges that are deep-rooted in the health system were identified. The suggested strategies to address them should be given due consideration not only to guide future mainstreaming efforts but also to facilitate health system strengthening.

## INTRODUCTION

Significant progress has been made in the last decade in controlling, eliminating, and eradicating neglected tropical diseases (NTDs). However, NTDs, remain major healthcare issues in many resource-limited settings, hindering progress to achieving Universal Health Coverage targets.^1^ NTDs affect ∼1.7 billion people worldwide, often those living in poverty with limited access to sanitation and health facilities and increased exposure to infectious vectors.^2^ NTDs are characterized by considerable morbidity and a significant reduction in quality of life.^2^^–^^4^

The WHO published “A roadmap for neglected tropical diseases (2021–2030),” in which accelerating programmatic actions against NTDs, intensifying cross-cutting approaches, and changing operating models and culture to facilitate country ownership were identified as the three pillars to support global efforts to control, eliminate, and eradicate NTDs.^1^ Mainstreaming NTDs interventions into national health systems was one of the proposed cross-cutting approaches. The terms “mainstreaming” and “integration” have been used interchangeably in the past to describe the assimilation of healthcare interventions into the general healthcare system. However, the WHO NTDs Roadmap uses “integration” to refer to the “grouping or packaging of several diseases to facilitate joint delivery of interventions through a common platform,” whereas “mainstreaming” is used to refer to “the planning and delivery of interventions against NTDs through the national health system infrastructure.”

In line with the WHO Roadmap, Ethiopia, one of the countries most affected by NTDs,^5^ is striving to mainstream NTDs control activities within the primary healthcare (PHC) system. The two major strategic recommendations outlined in the Third National NTDs Strategic Plan (2021–2025)^6^ and the Sustainability Action Plan for NTDs Control, Elimination, and Eradication (2021–2025)^7^ include strengthening the integration and linkage of NTDs programs with other healthcare programs and mainstreaming the national NTDs program activities into the national health system to ensure sustainability. However, there is a lack of evidence on managing the transition from a vertical setting to a mainstream healthcare delivery system in a real-world setting.

The aim of this study is to identify challenges and map potential strategies to mainstream campaign interventions against NTDs at each component of the health system guided by the Primary Health Care Performance Initiative (PHCPI) framework (https://improvingphc.org/). The WHO recommends five core strategic interventions to accelerate the prevention, control, elimination, and eradication of NTDs: 1) innovative and intensified disease management, 2) preventive chemotherapy, 3) vector control, 4) veterinary public health, and 5) the provision of safe water, sanitation, and hygiene.^1^ In this study, we used a participatory ranking methodology (PRM) through consultative workshops with purposively selected stakeholders from each health system structure to identify, rank, and contextualize challenges and strategies to mainstreaming campaign interventions against NTDs, which were then synthesized using the PHCPI framework. This was achieved by focusing on mainstreaming preventive chemotherapy, morbidity management, and disability prevention.

## MATERIALS AND METHODS

### Study context.

#### Country profile.

Ethiopia is the second most populous country in Africa, with a projected population of 126.5 million in 2024.^8^ The country is divided into 12 regions and two municipal administrations. The regions are further divided into zones, and the zones are divided into districts. The smallest administrative unit is the village (Kebele), with an average population of 5,000. Healthcare delivery management in Ethiopia is decentralized to the Regional Health Bureau, Zonal Health Departments, and Woreda (district) Health Offices at their respective levels. The healthcare system operates on a three-tier model (Supplemental Figure 1).^9^

1. Primary health care system: This district-level health system includes five satellite health posts serving an average village population of ∼5,000, a health center serving between 15,000 and 25,000 people, and a primary hospital serving 60,000–100,000 people in rural areas. In urban settings, a health center serves ∼40,000 people on average.
2. Secondary health care system: General hospitals serve 1–1.5 million people.
3. Tertiary health care system: Specialized hospitals cover a population of 3.5–5 million.

Ethiopia’s healthcare system is also classified into PHC supported by the Health Extension Program (HEP) and hospital-based services.^9^ The primary healthcare units are the main source of healthcare services for the rural community.^9^ The HEP is led by the health extension workers (HEWs), women who have received at least 1 year of full-time training. The HEP offers 18 primary care packages for family health, health promotion and disease prevention, hygiene, and environmental sanitation.^10^ However, the HEWs are also overwhelmed by a wide range of tasks from various healthcare programs. Within the HEP scope, the Health Development Army (HDA) program was launched in 2011. The HDAs were volunteers who were also early adopters of best hygiene and health practices and had completed the “model family” training. The HDA engages a group of households in their communities (one in five) to discuss key health issues and support the HEWs with tasks, such as community mobilization, household visits, and immunization and mass drug administration (MDA) campaigns.^11^ In addition to the government health system, local and international partners and the private sector play significant roles in Ethiopia’s health service delivery.

### NTDs program.

The Ministry of Health of Ethiopia, in partnership with its development and implementing partners, has made significant progress in reducing the burden of NTDs in the country. Ethiopia has prioritized nine NTDs as diseases of public health concern. These are trachoma, soil-transmitted helminths (STH), schistosomiasis, lymphatic filariasis (LF), onchocerciasis, dracunculiasis, leishmaniasis, podoconiosis, and scabies. Diseases targeted for preventive chemotherapy include trachoma, onchocerciasis, LF, STH, and schistosomiasis. Since 1994, the start of the guinea-worm disease eradication program, the Ministry of Health of Ethiopia and its partners distributed millions of treatments as part of the preventive chemotherapy through MDA for the five NTDs, provided morbidity management and disability prevention (MMDP) across the country, engaged with partners to improve access to water, sanitation, and hygiene, and conducted hundreds of disease prevalence and program impact surveys to guide programmatic decisions. The Third National NTDs Strategic Plan (2021–2025) aims to build a sustainable, resilient, high-quality, and equitable NTDs program that is fully mainstreamed into the national health system.^6^ Some of the NTDs program activities are already mainstreamed into the health system building blocks. For example, pharmaceutical products and other logistics management systems for the NTDs program are fully mainstreamed into the pharmaceutical and logistics management system. This includes drug logistics, storage, distribution, recording, and reporting. In addition, the NTDs program planning and reporting system has been partially mainstreamed into the National Health Information System. Among the 177 indicators included in the Health Management Information System, eight are for the NTDs.^12^

### Study design and participants.

A modified PRM was used, involving consultative stakeholder workshops to identify, rank, and contextualize challenges and strategies to mainstream NTDs campaign interventions in Ethiopia.^13^ Two separate consultative workshops were conducted with purposively selected stakeholders, including NTDs program leaders from the government and partners, PHC workers, and community leaders and volunteers. The first workshop was conducted in Adama town with national- and regional-level NTDs program leaders from the government and implementing partners, whereas the second was conducted in Shashemene Town with zonal and district health department officers, PHC facility heads, HEWs, community leaders, and volunteers.

### Data collection.

At the start of the workshops, the research protocol, objectives of the consultative workshop, and key terminologies were introduced and defined. “NTDs campaign interventions” in this study context were defined as “time-bound, intermittent activities against NTDs conducted to expediently fill service delivery gaps or provide surge coverage.”^14^ “Mainstreaming” was defined as “the extent, pattern, and rate of adoption and eventual assimilation of NTDs campaign interventions into each of the critical functions of a health system so that the primary health care system can independently plan, implement and report NTDs campaign activities.” Participants were divided into groups to discuss and identify (pile), rank, and provide contextualized meaning (account) for challenges and strategies related to mainstreaming NTDs campaign interventions.

#### Challenge piling.

Participants compiled (discussed and identified) 1) NTDs campaign interventions that could be mainstreamed in the current national NTDs program ecosystem and 2) current and future implementation challenges that hinder the mainstreaming of these NTDs campaign interventions. The participants were asked to list challenges individually. Then, the group leaders, participants chosen by each group to facilitate the discussion, invited each member to briefly state one item on their list until all challenges had been presented. These were compiled in an Excel sheet that was in full view of the group. Then the group discussed providing meaning to each item by clarifying, simplifying, and organizing. Some items were combined logically after the discussion and after a final consensus was reached.

#### Challenge ranking.

Participants within their groups were asked to individually rank the challenges identified on a scale of 1–10 based on 1) the importance of addressing the challenge for mainstreaming, which was characterized by the extent of benefit to be accrued from addressing the challenge, and 2) the feasibility of addressing the challenge. Participants were asked to consider 1) the resource requirement for addressing the challenge and 2) whether it is possible to address the challenge with the available technology, knowledge, and resources.

#### Challenge contextualizing.

Each group was asked to present their “account” to the general statements provided in the piling and ranking stage of the exercise to provide insight based on local contexts. The accounts stated by each group were then documented by the workshop notetakers and used in the discussion section of this manuscript to add meaning to the findings and build on contextualized knowledge of the subject.

#### Strategy piling.

This was performed in groups to identify potential solutions to address the ranked challenges. Participants individually identified possible solutions for each ranked challenge, and these were then discussed in the groups, and a final list was prepared.

#### Strategy ranking.

The strategies identified by each group were then ranked by the participants based on importance and feasibility. They were each scored for eight separate parameters (Table 1), with scores ranging between 0 and 10 (0 = very difficult to implement and less important; 10 = extremely easy to implement and very important).

**Table 1:** Mainstreaming strategy prioritization parameters

Parameter	Parameter Description
Importance	Importance of implementing the proposed strategy to mainstream the interventions
Health system/implementer willingness	The attitude and willingness of stakeholders at different levels to implement the proposed solution
Health system/implementer capacity	Capacity of the current PHC health system to implement the proposed strategy and mainstream the activity without external support
Implementation feasibility	Ease of implementing the solution and mainstreaming the activity
Financial feasibility	The health system’s financial capacity to implement the proposed strategy without external support
Logistics feasibility	Capacity to handle logistics issues related to implementing the proposed strategy
Efficiency	Capacity to implement the proposed strategy within the specified time, budget, and desired outcome
Acceptability	Acceptability of the proposed solution/strategy by the different stakeholders, government, community, partners, etc.

#### Strategy contextualizing.

The ranked strategies were presented and discussed to provide contextual meaning. These were documented by the workshop notetaker and used in the discussion section of this manuscript to unpack the recommended strategies.

#### Mainstreaming timeline.

Participants estimated the time required in years to partially or fully implement each strategy and thereby mainstream the NTDs campaign interventions into each function of the health system.

## STATISTICAL ANALYSES

**Figure 1.**
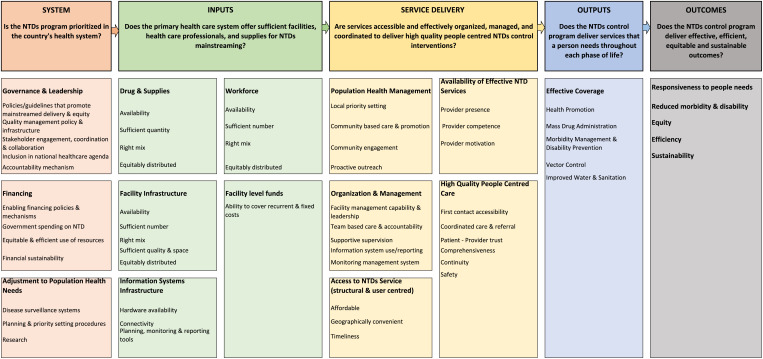
Primary Health Care Performance Initiative framework adapted for neglected tropical diseases control programs.

The ranking for each challenge and its solution was compiled and analyzed. Descriptive summary statistics were calculated in MS Excel (Microsoft Corp., Redmond, WA) to rank the challenges and solutions, the frequency of their citation, and the estimated time to address them. A thematic analysis was conducted to categorize and prioritize challenges and their solutions guided by the PHCPI framework modified for NTDs (Figure 1). This framework is designed to improve PHC services in low- and middle-income countries (LMICs) through the measurement and tracking of key performance indicators.^15^^,^^16^ The framework describes the critical components of a strong PHC system and guides programs and countries on what should be measured to inform and drive efforts to improve PHC by allowing them to identify strengths and challenges that need attention in their PHC system.^15^^,^^17^ Similarly, this framework was adapted to guide the data collation and synthesis of NTDs mainstreaming challenges and strategies to address them, organized under the following critical components of the health system: “system,” “inputs,” “service delivery,” “outputs,” and “outcomes.”

### Thematic analysis.

Challenges and solutions identified in the workshops were merged to produce a list of NTDs campaign interventions mainstreaming challenges and strategies. These were thematically categorized, and the frequency with which the challenges and the solutions were cited in the workshops are presented. For instance, challenges related to “shortage of primary health care worker,” “shortage of human resources,” and so on were categorized under the theme “workforce shortage,” whereas “low planning skills,” “poor competency,” and so on were categorized under “workforce competence gap.” Solutions provided to address workforce shortages, such as “recruit enough human resources,” “train and deploy HEWs,” “placement of competent finance personnel,” and so on, were categorized under the theme “deploy sufficient and competent workforce,” whereas solutions provided to address competence related to challenges such as “training,” “experience sharing,” “capacity building,” and so on were categorized under the theme “continuous on-the-job training to improve workforce competence.”

The thematically categorized mainstreaming challenges and solutions were categorized under each critical domain of the PHC system (system, inputs, service delivery, outputs, and outcomes) and their subdomains.^15^ The system domain has three subdomains, including “governance and leadership,” “financing,” and “adjustment to population health needs”; the inputs domain has five subdomains, including “drugs and supplies,” “facility infrastructure,” “information system infrastructure,” “workforce,” and “facility level funds”; the service delivery model has five subdomains, including “access to services,” “availability of effective services,” “high quality people centered care,” “service organization and management,” and “population health management”; the outputs domain has one subdomain, “effective coverage”; and the outcomes domain has five subdomains, including “responsiveness to people’s need,” “reduced morbidity and disability,” “equity,” “efficiency,” and “sustainability.” Accordingly, for example, “workforce shortage” was categorized under the “inputs” domain and “workforce” subdomain, whereas “workforce competence gap” was categorized under the “service delivery” domain and “availability of effective services” subdomain.

### Ranking and prioritization.

Each challenge was scored separately for its importance and the feasibility of addressing it. The two scores were separately summed up and divided by the number of participants to get an average score. The mean of these average scores for importance and feasibility were used to rank the challenges. Similarly, the score out of ten for each strategy (solution) in terms of the eight corresponding prioritization parameters (Table 1) was separately summed up and divided by the number of participants to get an average score. Then, the mean of the average score for the eight parameters was used to prioritize the strategies. The list of challenges and strategies to address them from the two consultative workshops were further reviewed for the frequency of citation by two experts (AH and EH). Duplicate challenges and strategies were then consolidated into a single final list, and the mean of their average scores was used for the ranking and prioritization.

### Implementation time.

The time estimated by the participants to partially or fully implement each strategy cited to address the mainstreaming challenges was separately summed and divided by the number of participants to compute the average time required for implementation. Then, the median time to implement the strategies partially and fully was computed. Strategies requiring shorter and longer times for partial and full implementation were identified.

## RESULTS

The two consultative workshops were conducted in November 2021. A total of 33 stakeholders from national, regional, district, and PHC levels participated, with the majority being district and PHC level workers (66.7%; Table 2). The participants identified 73 challenges and 185 strategies to address them, aimed at mainstreaming preventive chemotherapy and MMDP campaign interventions into the PHC system.

**Table 2:** Consultative workshop participants

Participants’ Institutions and Levels	Male	Female	Total
Ministry of Health	3	–	3
Implementing partners^†^	3	–	3
Regional health bureaus^‡^	3	–	3
Zonal health departments^§^	2	–	2
District health offices^§^	8	–	8
Health centers^§^	1	1	2
Health posts (health extension workers)^§^	–	6	6
Village leaders and health development army*^§^ members	4	2	6
Total	24 (72.7%)	9 (27.3%)	33

* A women-centered community movement organized in groups of six (one to five) representing the six nearest households (one leader and five members) mainly aimed at improving maternal health outcomes.

^†^ The END Fund, International Trachoma Initiative, National Podoconiosis Action Network.

^‡^ Amhara Regional State Health Bureau, Oromia Regional State Health Bureau, Southern Nations Nationalities and People Regional State Health Bureau.

^§^ From Oromia and Southern Nations Nationalities and People Regional States.

### Challenges.

#### Frequency.

**Figure 2.**
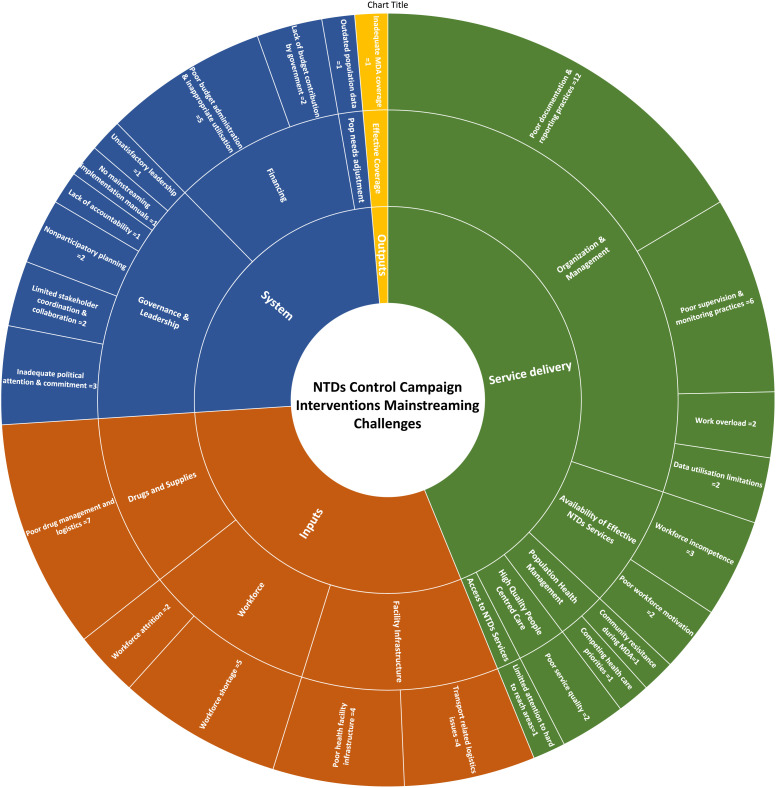
Summary of mainstreaming challenges (*N* = 73) identified by stakeholders (*N* = 33), categorized by the modified Primary Health Care Performance Initiative framework.

**Figure 3.**
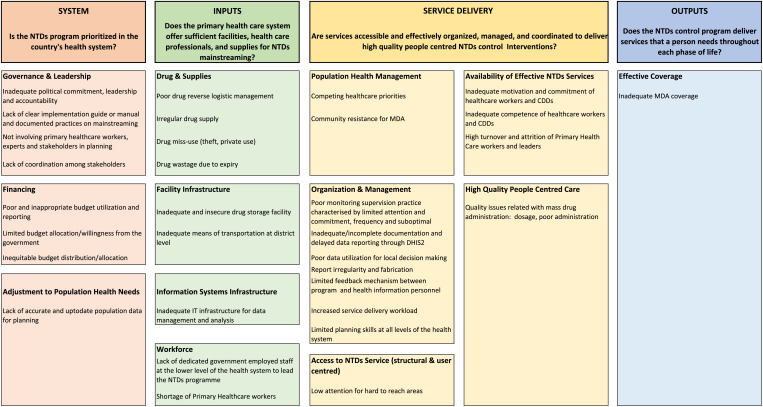
Consolidated neglected tropical diseases campaign intervention mainstreaming challenges (*n* = 32).

The identified mainstreaming challenges are summarized in Figure 2 based on the modified PHCPI framework. Among the 73 challenges, 32 (43.8%) were related to service delivery, 22 (30.1%) were related to inputs, 18 (24.7%) were related to systems, and 1 (1.4%) was related to outputs. The majority of challenges within the “service delivery,” “inputs,” and “system” domains were related to organization and management, workforce, and governance and leadership, respectively. The five most frequently cited challenges were poor data documentation and reporting practices (cited 12 times), poor drug management and logistics (cited seven times), weak supportive supervision and monitoring practices (cited six times), health workforce shortage (cited five times), and poor budget administration and inappropriate utilization (cited five times). After reviewing for duplication, the list of challenges was consolidated into 32, as shown in Figure 3. Among these, more than half (14) were “service delivery” challenges, with the majority (seven) related to improper NTDs service organization and management. Of the nine “inputs” related challenges, four were on drugs and supplies. Of the eight “system” challenges half (four) were on governance and leadership. The three key financing challenges under the system domain were inappropriate budget administration, utilization, and reporting; lack of government budget contribution; and inequitable budget distribution. The “outputs”-related challenge was on inadequate MDA coverage.

#### Ranking.

The top ten ranked challenges are presented in Table 3. Four were related to “service delivery,” four were related to “system,” and two were related to “inputs.” The three top-ranked challenges were 1) poor monitoring and supportive supervision practices (mean score 9.0), which were characterized by limited attention, commitment, frequency, and suboptimal quality; 2) inadequate or incomplete data documentation and delayed reporting (mean score 8.6); and 3) poor drug reverse logistics management (mean score 8.2). Drug reverse logistics management refers to the return of unused drugs from the PHC units to the district health offices after the MDA implementation for redistribution in the next MDA cycle. The complete prioritization for the 32 mainstreaming challenges is presented in Supplemental Table 1.

**Table 3:** Top ten ranked challenges for NTDs campaign interventions mainstreaming according to PHCPI and associated strategies

PHCPI Domain	PHCPI* Subdomain	Challenge Rank	Challenge Ranking Score (out of 10)	Identified Challenges	Strategy (solutions)	Strategy Ranking Score (out of 10)
Service delivery	Organization and management	1	9	Weak monitoring and supportive supervision practices characterized by limited attention, commitment, and frequency and suboptimal quality	Develop and apply standard and integrated monitoring and supportive supervision planning and implementation tool	8.4
					Build the capacity of supervisors to conduct integrated monitoring and supervision	7.7
					Establish motivational incentive package to maximize supervision and monitor productivity and quality	7.7
		2	8.6	Inadequate/incomplete data documentation and delayed reporting through DHIS2	Build the capacity of primary and district-level personnel and health information technicians	7.9
					Close follow-up, review, and feedback on report content and timings by program management team at all levels	7.9
		9	7.5	Limited feedback mechanism between program and health information personnel	Develop and implement standardized and user-friendly feedback tool	8.1
		7	7.7	Poor data utilization for local decision-making	Building local capacity for data analysis and interpretation	7.6
Inputs	Drugs and supplies	3	8.2	Poor drug reverse logistic management	Active monitoring and follow-up of reverse drug management by personnel at all levels of the health system	7.4
					Delegation of personnel that can effectively lead drug reverse logistics at the district level	6.7
					Early and timely initiation of drug reverse logistic management activities	6
		4	7.9	Irregular drug supply	Effective micro planning, early drug requests, and speedy decision-making	7.8
					Establish effective and coordinated drug transfer system at all levels of the health system	7.7
System	Financing	5	7.9	Poor budget administration and reporting and inappropriate utilization	Placing competent finance personnel at all levels and delivering training on financial management and reporting	7.7
					Establish transparent financial management and reporting system and conduct regular and structured monitoring of budget utilization	7.3
					Take immediate corrective action on inappropriate budget users	6.7
	Governance and leadership	6	7.8	Inadequate political commitment and accountability	Delegation of activities and responsibility with authority to enhance decision-making ability	7.9
					Develop and implement strong advocacy plan targeting political leaders at all levels of the health system	7.8
System	Governance and leadership	8	7.6	Lack of clear implementation guide or manual and documented practices on mainstreaming	Document promising practices and develop and implement context-specific mainstreaming implementation manual	7.3
					Include NTDs control activities in the deliverables and evaluation agenda of political leaders	7.7
System	Adjustment to population health needs	10	7.5	Lack of accurate and up-to-date population data for planning	Use population data estimates from National and Regional policy and plan offices	8.4

DHIS2 = District Health Information Software v2; NTDs = neglected tropical diseases; PHCPI = Primary Health Care Performance Initiative.

### Strategies.

#### Frequency.

**Figure 4.**
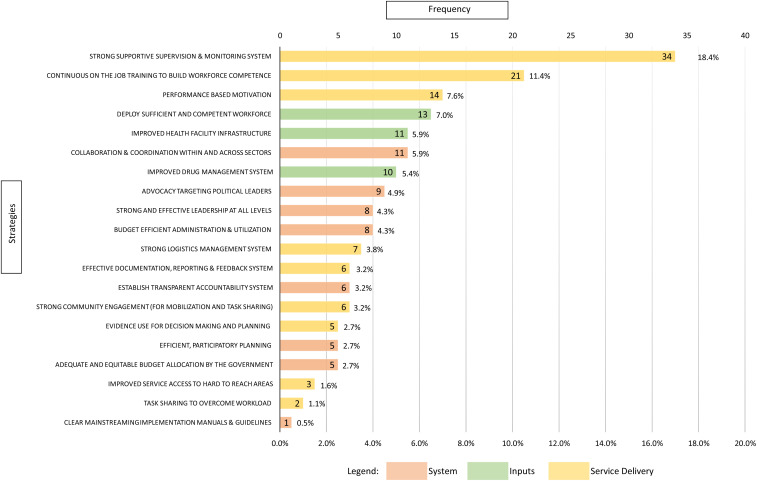
Summary of strategies identified by stakeholders (*N* = 33) to address mainstreaming challenges (*N* = 185).

The strategies identified to address the 73 challenges are summarized in Figure 4. Overall, “service delivery”-related strategies constituted 53% (98/185) of the total cited strategies, whereas “system”- and “inputs”-related strategies constituted 28.6% (53/185) and 18.4% (34/185), respectively. The three most frequently cited strategies were related to the “service delivery” domain of the PHCPI: establishing a strong and supportive supervision and monitoring system (cited 34 times), continuous on-the-job training to build workforce competence (cited 21 times), and a performance-based motivation system (cited 14 times). After review for duplication, the 185 strategies were consolidated into 56. These are presented in Supplemental Table 2, along with the respective challenges they are cited to address.

#### Ranking.

The top ten ranked strategies linked with the challenges they are cited to address are presented in Table 4. Four of these strategies address “service delivery” challenges, whereas three address “input” challenges, two address “system” challenges, and one addresses an “outputs” challenge. The top three ranked strategies were 1) implementing standardized and evidence-based budget distribution criteria (mean score 8.5) to address inequitable budget distribution, 2) establishing an effective first in, first out drug management system (mean score 8.5) to prevent drug wastage due to expiry, and 3) applying standardized and integrated monitoring and supervision tool (mean score 8.4) to improve service organization and management.

**Table 4:** Top ten ranked strategies to address neglected tropical diseases campaign interventions mainstreaming challenges according to PHCPI framework, and associated challenges

Strategy Rank	Strategy Score (out of 10)	Strategy (solutions)	Challenges the Strategies Are Linked To	Challenge Score	PHCPI Subdomain	PHCPI Domain
1	8.5	Implement standardized and evidence-based budget distribution criteria (matrix)	Inequitable budget distribution/allocation	7.1	Financing	System
2	8.5	Establish effective first in, first out system	Drug wastage due to expiry	6.7	Drugs and supplies	Inputs
3	8.4	Develop and implement standard and integrated monitoring and supportive supervision planning and implementation tool	Poor monitoring and supportive supervision practice characterized by limited attention and commitment, frequency, and suboptimal quality	9	Organization and management	Service delivery
4	8.4	Use population data estimates from National and Regional policy and plan offices	Lack of accurate and up-to-date population data for planning	7.5	Adjustment to population health needs	System
5	8.3	Involve key religious and community leaders in MDA implementation	Community resistance to MDA	6.9	Population health management	Service delivery
6	8.2	Close monitoring and follow-up	Report irregularities and fabrication	7.4	Organization and management	Service delivery
7	8.1	Develop and implement standardized and user-friendly feedback tool	Limited feedback mechanism between program and health information personnel	7.5	Organization and management	Service delivery
8	8.1	Strict regulation and control through drug tracking and accountability	Drug misuse (theft, private use)	6.7	Drugs and supplies	Inputs
9	8.1	Timely inventory, reporting, and transfer of drugs and supplies	Drug wastage due to expiry	6.7	Drugs and supplies	Inputs
10	8	Implement strong community mobilization and awareness program	Inadequate MDA coverage	7.1	Effective coverage	Outputs

MDA = mass drug administration; PHCPI = Primary Health Care Performance Initiative.

#### Implementation time.

The median time to partially implement the strategies cited to address the challenges was 2.4 years (IQR, 2.3–2.7), whereas the median time for full implementation was 3.4 years (IQR, 2.9–3.9). The strategies for “inputs” related challenges were scored to take a longer time for both partial (median 2.5 years; IQR 2.4–2.9) and full implementation (median 3.9 years; IQR 3.0–4.2), whereas the strategies for “system” challenges were scored to take shorter time for both partial (median 2.4 years; IQR 2.3–2.7) and full-scale implementation (median 3.0 years; IQR 3.0–4.1). The delegation of personnel to effectively lead drug reverse logistic management at the district level was given the shortest average time for both partial and full implementation at 2.0 years and 2.6 years, respectively. On the other hand, establishing a conducive drug storage facility at the health post level, either by constructing new facilities or renovating existing ones, was cited as taking a much longer time for both partial (4.3 years) and full (5.4 years) implementation.

## DISCUSSION

Multifaceted mainstreaming challenges and their solutions were identified for each major component of the health system. The key mainstreaming challenges were related to organization and management within the service delivery domain, drug and supply logistics within the inputs domain, and financing, governance, and leadership within the system domain of the PHCPI. The Ministry of Health of Ethiopia and its partners can use the evidence generated by this study to identify the tools, training, infrastructure, and logistics support needed at each building block of the health system to facilitate the mainstreaming process. In the following sections, we have contextualized the findings under the three critical components of the health system, “service delivery,” “inputs,” and “system,” and suggested how they can be used to inform the national program mainstreaming efforts.

### Service delivery.

The challenges identified in mainstreaming NTDs into the healthcare system are primarily related to inadequate organization and management at the point of service delivery. Poor documentation, reporting, monitoring, and supportive supervision practices were the most frequently cited and top-ranked challenges. Building the capacity of primary healthcare workers and information technicians, establishing a review and feedback system on report content and timing, and continuous monitoring and quality improvement systems are essential. Supportive supervision helps to improve workforce competence and motivation.^17^ Studies from LMICs have demonstrated that supportive supervision improves the quality and productivity of NTDs control and other healthcare services.^18^^–^^20^

The workforce competence gap and lack of motivation were among the challenges anticipated to hinder the mainstreaming efforts. A study in Nigeria found that mainstreaming may discourage implementers unless significant but gradual and coordinated changes are made in the health system.^21^ The availability of effective NTDs services and coverage depends on competence and motivation dimensions.^17^ Strategies identified to address these include continuous on-the-job training, experience sharing, establishing a system for mentoring, and regular supportive supervision. A small-scale study conducted in southern Ethiopia found that training PHC workers to detect and manage NTDs improved knowledge but did not lead to a practical skill difference compared with those with no training, implying that ongoing training and supportive supervision are required for PHC workers to develop meaningful competence on the detection, management, recording, and reporting of NTDs.^22^ Mainstreaming service delivery may impose a workload on PHC workers and have a long-term impact on motivation.^23^ The overwhelming burden on the HEWs is a good example. HEWs in Ethiopia are tasked with implementing a multitude of healthcare programs, some of which are above their competence level, suggesting systematic issues. Mainstreaming NTDs interventions may not only add to their overload but also their complexity. Task sharing by other cadres and continuous motivation strategies were recommended. The study in Nigeria suggested the need for government budget allocation, trust building, and robust and targeted training for effective mainstreaming.^21^

### Inputs.

The key “inputs”-related challenges and solutions were from the drug and supplies, workforce, and facility infrastructure subdomains. Poor drug management and logistics characterized by poor reverse logistics management, irregular drug supply, drug wastage due to expiry, and drug misuse were among the most frequently cited mainstreaming challenges. A poor drug reverse logistic management system would lead to drug misuse and expiry, and these in turn result in irregular drug stock management and an irregular supply system. Drug misuse and wastage cannot be left unaddressed, whether programs shift to a mainstream approach or not. Strict regulation and control mechanisms with clear accountability through drug tracking and the timely inventory, reporting, transfer, and disposal of expired and unserviceable drugs need to be in place. The lack or absence of essential drugs and supplies would simply mean no services. A study in Ethiopia has found a significant association between performing trachomatous trichiasis surgery and reported availability of consumables.^19^ Effectively transferring the logistics and accountability system used and supported by implementing partners such as the International Trachoma Initiatives for azithromycin into the regular health system logistics would be key in improving reverse logistics management and preventing drug wastage and misuse.

Workforce shortage puts pressure on the health system, contributing to service organization and management challenges, and compromises the availability of equitably accessible people-centered quality services. However, the workforce shortage is unlikely to be related to a lack of trained human resources, given the huge number of unemployed health workers in the country.^24^^,^^25^ An assessment conducted by the World Bank has identified fiscal constraints that limit the number of health jobs available, contributing to the high health workforce unemployment in Ethiopia.^24^ In addition to the lack of budget for remuneration, workforce attrition and its effect on inequitable distribution are likely contributors to the below-standard health workforce density in the country. Hence, training more PHC workers would not be a fitting solution. A more comprehensive strategy and reform involving increasing government funding for health and improving health workforce remuneration, career structure, management, and support systems needs to be used.^24^ Studies have previously demonstrated that living conditions and remuneration are important determinants of workforce retention in low-income settings.^18^^,^^19^^,^^26^

Addressing poor health facility infrastructure at the PHC level, including inadequate and insecure drug storage facilities, inadequate information technology (IT) infrastructure and human resources for data management, and a lack of transport facilities, requires intersectoral collaboration and long-term investment. The adequacy of health facility infrastructure will not only directly impact service delivery quality but is also linked with health workforce stability. Access to transport and IT infrastructure was linked with workforce retention in a previous study in Ethiopia.^19^

### System.

The key governance and leadership issues raised were inadequate political attention, commitment, and accountability; limited coordination and collaboration within and across sectors and stakeholders; and the exclusion of PHC workers and other key stakeholders from planning. These challenges and the strategies identified to address them were aligned with the recommendations in the WHO NTDs Roadmap, in which advocacy, stakeholder collaboration, and multisectoral action are identified as enablers for accelerated programmatic action.^1^ Achieving the 2030 NTDs elimination target through mainstreamed delivery would require ownership, commitment, and accountability from the in-country leadership. A systematic review of the integrated delivery of NTDs interventions identified governance among the key elements for success.^27^ Advocacy targeting political leaders at all levels of the health system is crucial to include NTDs control activities at the forefront of their agenda to improve domestic financing and accountability. It is important to get across the message that action against these diseases of poverty is core to achieving sustainable development goals.^1^

Mainstreaming campaign interventions in the health system alone is probably insufficient to achieve elimination and sustainability. Collaboration and coordination both within and between sectors are mandatory. A good example is the need to mainstream health education campaigns into the education sector. Coordination with other seemingly distant but key sectors, such as ministries of finance and communication, and engaging them actively from planning to evaluation is likely to determine mainstreaming success. As shown in this study, establishing a stakeholder coordination team and implementing a standardized stakeholder collaboration or engagement guide and accountability framework would be useful to facilitate, successfully manage, and benefit from the collaborations. Aligned with the rationale for our phased implementation research, the lack of a clear implementation mainstreaming manual or documented preferred practices was one of the top-ranked mainstreaming challenges. Implementation research that can generate practical evidence and feed into mainstreaming technical manuals and policies needs to be encouraged.

The key financing challenges identified were poor budget administration, inappropriate utilization, and a lack of adequate government financing for NTDs. One recommendation of the WHO NTDs roadmap is that countries actively integrate NTDs interventions in their national and local government health and budget plans proportionate with the magnitude of NTDs.^1^ The key goal of mainstreaming is sustainability, and this would not be achieved without domestic financing, mainly through government financial contributions. Ethiopia’s government health expenditure in 2021 was ∼5.4% of the gross domestic product and 7.1% of the general government expenditures.^28^ This equates to $26 per capita, compared with the $86 recommended by WHO for LMICs.^24^^,^^28^ In 2021, out of the total Ethiopia health spending, 37%, 30.5%, and 28.3% come from out-of-pocket spending, government health spending, and external aid, respectively.^28^ The WHO states that less than 1% of domestic expenditure on health would achieve the 2030 NTDs elimination targets.^1^ NTDs are associated with poverty and are often proxy for inequality.^2^^,^^29^ Thus, in addition to the health sector plan, government poverty alleviation programs need to include a budget for NTDs. Poor budget administration and inappropriate use will work against the whole purpose of mainstreaming, which is creating a self-sustaining program. Workforce capacity-building plans and activities need to include finance personnel to address limitations on budget administration competence. Standardized evidence-based budget distribution criteria need to be established for equitable budget distribution. Transparent and accountable financial management and reporting systems with strict monitoring of budget utilization need to be in place to prevent the inappropriate use of available budgets and ultimately contribute to successful mainstreaming.

### Strengths and limitations.

To the best of our knowledge, this is the first study to explore NTDs campaign intervention mainstreaming challenges guided by the PHCPI framework. The challenges and strategies identified are linked to the key components of the health system, facilitating easy understanding, role allocation, and implementation at the different levels of the health system. The methodology used in this study can be replicated to generate similar context-specific data in other settings, particularly where resources are scarce. However, this study has several limitations. The people involved in the stakeholder workshop were limited in number, geography, and mix. Two people were recruited from health centers, and none were recruited from primary or district-level hospitals. The regional-, zonal-, district-, and PHC-level participants were only from two out of the ten regions in Ethiopia. Other key stakeholders that directly influence campaign intervention mainstreaming, such as those from the Ministry of Finance and the Ethiopia Pharmaceutical Supply Agency, were not involved. The second consultative workshop was conducted with participants ranging from zonal health officers to community volunteers. It is possible that the contribution of some of the participants from the lower tier of the health system was inhibited because of the presence of “their supervisors.” Having more HEWs in the discussions who could speak freely without supervisors being present would have been useful. It is also likely that some of the questions posed in the consultative workshop may not have been adequately understood by participants from the lower tier of the health system. The HEWs were not given the opportunity to talk about how they feel about the increased workload and what they need to be effective. However, we have conducted a separate follow-on study, in which semi-structured interviews were used with HEWs, HDAs, and other PHC cadres to explore several concepts related to mainstreaming. The qualitative insights that were identified for each challenge and strategy during the group discussion and presentations were not adequately reflected in the results because of the quantitative nature of the ranking exercise. This, however, is one of the notable limitations of the PRM. To address this, in the discussion section, guided by the PHCPI framework, we tried to unpack the seemingly “general statement” of challenges and strategies, providing contextual meaning based on the insights provided during the consultive workshops. The implementation timeline provided by the participants to partially and fully mainstream campaign interventions cannot be supported by evidence or tested experience. The focus of this study has been on NTDs campaign intervention mainstreaming efforts; therefore, challenges related to non-campaign-based interventions were excluded by design. However, the challenges identified are overarching and are likely to impact the mainstreaming of any NTDs intervention, regardless of its mode of delivery. The results presented in this study are specific to the Ethiopian health system, limiting its generalizability. However, some of the findings, with some adaption, can be used to inform mainstreaming efforts in settings with similar healthcare structures.

## CONCLUSION

Country ownership and the mainstreaming of the NTDs program are reflected in its commitments toward governance, leadership, and financing. Successful program mainstreaming will require government financial contribution, without which it would be unrealistic to expect a fully fledged transition. The transition may start with a genuine partnership with international partners in which the government invests in key “system” and “input” functions, such as maintaining up-to-date population censuses, establishing efficient data management and supply chain systems, and addressing human resource-related issues, while the international partners focus on financing specific gaps, such as capacity building and field activities, through the strengthened health system. Otherwise, a lack of adequate government financial contribution for NTDs either in health or development programs means contributing further the neglect and inequality. Before fully embarking on mainstreaming, further assessments of the health system’s capacity and readiness should be conducted. This could be followed by developing a clear context-specific mainstreaming implementation tool and piloting it in a few districts.

## Supplemental Materials

10.4269/ajtmh.24-0261 Supplemental Materials
