# Supplementary material for: Challenges and Strategies for Mainstreaming Neglected Tropical Diseases Campaign Interventions in Ethiopia
Source: Am J Trop Med Hyg. 2024 Nov 26;112(2):467–78. doi: 10.4269/ajtmh.24-0261 (PMC11803666; doi:10.4269/ajtmh.24-0261)
Supplement: Supplemental Materials [file tpmd240261.SD1.pdf]

Supplemental Figure 1: Ethiopia Health Care System Structure

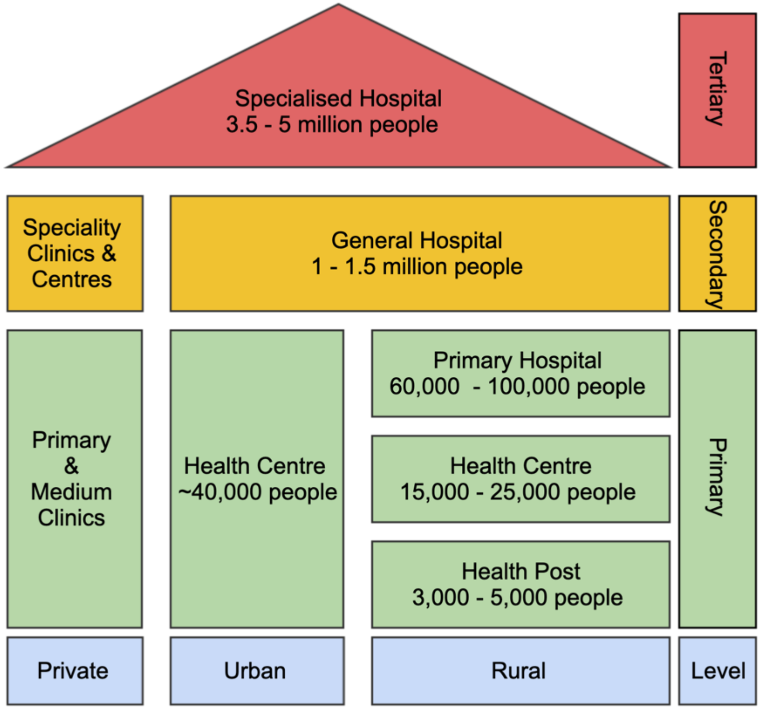

Supplementary Table 1: Ranking of the full list of the consolidated NTDs campaign intervention mainstreaming challenges

PHCPI domain	PHCPI subdomain	Challenge rank	Challenge ranking score (out of 10)	Identified challenges	Strategy (solutions)	Strategy ranking score (out of 10)
Service Delivery	Organization & Management	1	9	Poor monitoring and supportive supervision practice characterized by limited attention, commitment, frequency and suboptimal quality	Develop and apply standard and integrated monitoring and supportive supervision planning and implementation tool	8.4
					Build the capacity of supervisors on conducting integrated monitoring and supervision	7.7
					Establish motivational incentive package to maximize supervision and monitoring productivity and quality	7.7
		2	8.6	Inadequate/incomplete data documentation and delayed reporting through DHIS2	Build the capacity of primary and district level personnel and health information technicians	7.9
					Close follow up, review and feedback on report content and timings by programme management team at all levels	7.9
Inputs	Drugs & Supplies	3	8.2	Poor drug reverse logistic management	Active monitoring and follow up of reverse drug management by personnel at all levels of the health system	7.4
					Delegation of personnel that can effectively lead drug reverse logistics at the district level	6.7
					Early and timely initiation of drug reverse logistic management activities	6
		4	7.9	Irregular drug supply	Effective micro planning, early drug request and speedy decision making	7.8
					Establish effective and coordinated drug transfer system at all levels of the health system	7.7
Systems	Financing	5	7.9	Poor budget administration, reporting and inappropriate utilization	Placing competent finance personnel at all levels and delivering training on financial management and reporting	7.7
					Establish transparent financial management and reporting system and conduct regular and structured monitoring on budget utilization	7.3

					Take immediate corrective action on inappropriate budget users	6.7
	Governance & Leadership	6	7.8	Inadequate political commitment and accountability	Delegation of activities and responsibility with authority to enhance decision making ability	7.9
					Develop and implement strong advocacy plan targeting political leaders at all levels of the health system	7.8
Service Delivery	Organization & Management	7	7.7	Poor data utilization for local decision making	Building local capacity for data analysis and interpretation	7.6
Systems	Governance & Leadership	8	7.6	Lack of clear implementation guide or manual and documented practices on mainstreaming	Document promising practices and develop and implement context specific mainstreaming implementation manual	7.3
					Include NTDs control activities in the deliverables and evaluation agenda of political leaders	7.7
Service Delivery	Organization & Management	9	7.5	Limited feedback mechanism between programme and health information personnel	Develop and implement standardized and user-friendly feedback tool	8.1
Systems	Adjustment to Population Health Needs	10	7.5	Lack of accurate and uptodate population data for planning	Use population data estimates from National and Regional policy and plan offices	8.4
Systems	Governance & Leadership	11	7.4	Not involving primary healthcare cadres, experts and stakeholders in planning	Establish participatory planning by involving district and PHCU cadres and other key stakeholders in the planning process	7.9
Inputs	Facility Infrastructure	12	7.4	Inadequate and insecure drug storage facility at Health Post level	Works closely with village leaders to assign security guards for health posts	7.0
					Establish a conducive drug storage facility at Health posts either by constructing new or renovating existing ones	6.8
Service Delivery	Organization & Management	13	7.4	Report irregularities and fabrication	Close monitoring and follow up	8.2
Inputs	Information Systems Infrastructure	14	7.4	Inadequate IT infrastructure (Computer, Tablets, Internet) for data management and analysis	Procure and avail IT infrastructure	7.2
Service Delivery	High Quality People Centred Care	15	7.3	Quality issues related with mass drug administration: dosage, poor administration	Adequate training for primary healthcare cadres	7.6
					Targeted supportive supervision	7.3

	Population Health Management	16	7.2	Competing healthcare priorities	Careful planning of activities for integrated execution	7.6
Systems	Financing	17	7.1	Limited budget allocation/willingness from the government	Develop and implement strong advocacy plan targeting political leaders at all levels of the health system	7.8
Outputs	Effective Coverage	18	7.1	Inadequate MDA coverage	Implement strong community mobilization and awareness programme	8
					Strong monitoring and evaluation	7.7
					Strong district level advocacy to buy-in political support	7.4
Service Delivery	Availability of Effective NTDs Service	19	7.1	Inadequate motivation and commitment of healthcare workers and CDDs	Develop and implement standardized motivation and recognition package for health workers and CDDs	7.6
					Improve healthcare workers career structure	7
					Performance based incentives	6.7
Inputs	Workforce	20	7.1	Lack of dedicated government employed staff at the lower level of the health system to lead the NTDs Programme	Revise the HR structure and assign dedicated NTDs focal person at PHCU	7.6
Systems	Financing	21	7.1	Inequitable budget distribution/allocation	Implement standardized and evidence-based budget distribution criteria (Matrix)	8.5
Service Delivery	Availability of Effective NTDs Service	22	7	Healthcare workers and CDDs competence gap	Establish a system for mentoring, experience sharing and regular supportive supervision	7.1
Service Delivery	Access to NTDs Services	23	7	Limited attention to hard-to-reach areas	Implement clear prioritization criteria based on disease burden	6.1
Systems	Governance & Leadership	24	6.9	Lack of coordination among stakeholders	Establish stakeholder coordination team	8
					Design standardized term of reference or guide for collaboration	7.9
					Schedule regular planning and monitoring meeting	7.6
Service Delivery	Availability of Effective NTDs	25	6.9	High turnover and attrition of Primary Health Care workers and	Establish performance-based appraisal	7.5

	Service			leaders	Create conducive working environment	7.2
					Establish effective handover mechanisms	7.4
	Population Health Management	26	6.9	Community resistance for MDA	Involve key religious and community leader in MDA implementation	8.3
					Improve community awareness	7.4
Inputs	Workforce	27	6.8	Shortage of Primary Healthcare Cadres	Trainee and deploy more Primary Healthcare Workers, HEWs	7.4
					Revise health worker deployment system to ensure equitable distribution	7.3
Service Delivery	Organization & Management	28	6.8	Limited planning skills at all levels of the health system	Building the planning capacity of healthcare workers and leaders through training and experience sharing	7.4
Inputs	Drugs & Supplies	29	6.7	Drug miss-use (theft, private use)	Strict regulation and control through drug tracking and accountability	8.1
		30	6.7	Drug wastage due to expiry	Establish effective first in first out system	8.5
					Timely inventory, reporting and transfer of drugs and supplies	8.1
	Facility Infrastructure	31	6.7	Inadequate means of transportation at district level	Effective coordination to maximize efficient use of available transport means at the district level	7.8
					Adequate budget allocation for vehicle purchase at districts and PHC facilities	6.4
Service Delivery	Organization & Management	32	6.5	Increased service workload	Implement task sharing to ease workload	7.8

Supplemental Table 2: Ranking of the full list of the consolidated NTDs campaign intervention mainstreaming strategies

Rank	Strategy ranking score (out of 10)	Strategy (solutions)	Identified Challenges	Challenge ranking score (out of 10)	Subdomain	Domain
1	8.5	Implement standardized and evidence-based budget distribution criteria (Matrix)	Inequitable budget distribution/allocation	7.1	Financing	Systems
2	8.5	Establish effective first in first out system	Drug wastage due to expiry	6.7	Drugs & Supplies	Inputs
3	8.4	Develop and apply standard and integrated monitoring and supportive supervision planning and implementation tool	Poor monitoring and supportive supervision practice characterized by limited attention and commitment, frequency and suboptimal quality	9	Organization & Management	Service Delivery
4	8.4	Use population data estimates from National and Regional policy and plan offices	Lack of accurate and uptodate population data for planning	7.5	Adjustment to Population Health Needs	Systems
5	8.3	Involve key religious and community leader in MDA implementation	Community resistance for MDA	6.9	Population Health Management	Service Delivery
6	8.2	Close monitoring and follow up	Report irregularities and fabrication	7.4	Organization & Management	Service Delivery
7	8.1	Develop and implement standardized and user-friendly feedback tool	Limited feedback mechanism between programme and health information personnel	7.5	Organization & Management	Service Delivery
8	8.1	Strict regulation and control through drug tracking and accountability	Drug miss-use (theft, private use)	6.7	Drugs & Supplies	Inputs
9	8.1	Timely inventory, reporting and transfer of drugs and supplies	Drug wastage due to expiry	6.7	Drugs & Supplies	Inputs
10	8	Implement strong community mobilization and awareness programme	Inadequate MDA coverage	7.1	Effective Coverage	Outputs
11	8	Establish stakeholder coordination team	Lack of coordination among stakeholders	6.9	Governance & Leadership	Systems

12	7.9	Build the capacity of primary and district level personnel and health information technicians	Inadequate/incomplete documentation and delayed data reporting through DHIS2	8.6	Organization & Management	Service Delivery
13	7.9	Close follow up, review and feedback on report content and timings by programme management team at all levels	Inadequate/incomplete documentation and delayed data reporting through DHIS3	8.6	Organization & Management	Service Delivery
14	7.9	Delegation of activities and responsibility with authority to enhance decision making ability	Inadequate political commitment and accountability	7.8	Governance & Leadership	Systems
15	7.9	Establish participatory planning by involving district and PHCU cadres and other key stakeholders in the planning process	Not involving primary healthcare cadres, experts and stakeholders in planning	7.4	Governance & Leadership	Systems
16	7.9	Design standardized term of reference or guide for collaboration	Lack of coordination among stakeholders	6.9	Governance & Leadership	Systems
17	7.8	Effective micro planning, early drug request and speedy decision making	Irregular drug supply	7.9	Drugs & Supplies	Inputs
18	7.8	Develop and implement strong advocacy plan targeting political leaders at all levels of the health system	Inadequate political commitment and accountability	7.8	Governance & Leadership	Systems
19	7.8	Develop and implement strong advocacy plan targeting political leaders at all levels of the health system	Limited budget allocation/willingness from the government	7.1	Financing	Systems
20	7.8	Effective coordination to maximize efficient use of available transport means at the district level	Inadequate means of transportation at district level	6.7	Facility Infrastructure	Inputs
21	7.8	Implement task sharing to ease workload	Increased service workload	6.5	Organization & Management	Service Delivery
22	7.7	Build the capacity of supervisors on conducting integrated monitoring and supervision	Poor monitoring and supportive supervision practice characterized by limited attention and commitment, frequency and suboptimal quality	9	Organization & Management	Service Delivery
23	7.7	Establish motivational incentive package to maximize supervision and monitoring productivity and quality	Poor monitoring and supportive supervision practice characterized by limited attention and commitment, frequency and suboptimal quality	9	Organization & Management	Service Delivery
24	7.7	Establish effective and coordinated drug transfer system at all levels of the health system	Irregular drug supply	7.9	Drugs & Supplies	Inputs
25	7.7	Placing competent finance personnel at all levels and delivering training on financial management and reporting	Poor budget administration, reporting and inappropriate utilization	7.9	Financing	Systems

26	7.7	Include NTDs control activities in the deliverables and evaluation agenda of political leaders	Lack of clear implementation guide or manual and documented practices on mainstreaming	7.6	Governance & Leadership	Systems
27	7.7	Strong monitoring and evaluation	Inadequate MDA coverage	7.1	Effective Coverage	Outputs
28	7.6	Building local capacity for data analysis and interpretation	Poor data utilization for local decision making	7.7	Organization & Management	Service Delivery
29	7.6	Adequate training for primary healthcare cadres	Quality issues related with mass drug administration: dosage, poor administration	7.3	High Quality People Centred Care	Service Delivery
30	7.6	Careful planning of activities for integrated execution	Competing healthcare priorities	7.2	Population Health Management	Service Delivery
31	7.6	Develop and implement standardized motivation and recognition package for health workers and CDDs	Inadequate motivation and commitment of healthcare workers and CDDs	7.1	Availability of Effective NTDs Service	Service Delivery
32	7.6	Revise the HR structure and assign dedicated NTDs focal person at PHCU	Lack of dedicated government employed staff at the lower level of the health system to lead the NTDs Programme	7.1	Workforce	Inputs
33	7.6	Schedule regular planning and monitoring meeting	Lack of coordination among stakeholders	6.9	Governance & Leadership	Systems
34	7.5	Establish performance-based appraisal	High turnover and attrition of Primary Health Care workers and leaders	6.9	Availability of Effective NTDs Service	Service Delivery
35	7.4	Active monitoring and follow up of reverse drug management by personnel at all levels of the health system	Poor drug reverse logistic management	8.2	Drugs & Supplies	Inputs
36	7.4	Strong district level advocacy to buy-in political support	Inadequate MDA coverage	7.1	Effective Coverage	Outputs
37	7.4	Establish effective handover mechanisms	High turnover and attrition of Primary Health Care workers and leaders	6.9	Availability of Effective NTDs Service	Service Delivery
38	7.4	Improve community awareness	Community resistance for MDA	6.9	Population Health Management	Service Delivery
39	7.4	Trainee and deploy more Primary Healthcare Workers, HEWs	Shortage of Primary Healthcare Cadres	6.8	Workforce	Inputs

40	7.4	Building the planning capacity of healthcare workers and leaders through training and experience sharing	Limited planning skills at all levels of the health system	6.8	Availability of Effective NTDs Service	Service Delivery
41	7.3	Establish transparent financial management and reporting system and conduct regular and structured monitoring on budget utilization	Poor budget administration, reporting and inappropriate utilization	7.9	Financing	Systems
42	7.3	Document promising practices and develop and implement context specific mainstreaming implementation manual	Lack of clear implementation guide or manual and documented practices on mainstreaming	7.6	Governance & Leadership	Systems
43	7.3	Targeted supportive supervision	Quality issues related with mass drug administration: dosage, poor administration	7.3	High Quality People Centred Care	Service Delivery
44	7.3	Revise health worker deployment system to ensure equitable distribution	Shortage of Primary Healthcare Cadres	6.8	Workforce	Inputs
45	7.2	Procure and avail IT infrastructure	Inadequate IT infrastructure (Computer, Tablets, Internet) for data management and analysis	7.4	Information Systems Infrastructure	Inputs
46	7.2	Create conducive working environment	High turnover and attrition of Primary Health Care workers and leaders	6.9	Availability of Effective NTDs Service	Service Delivery
47	7.1	Establish a system for mentoring, experience sharing and regular supportive supervision	Healthcare workers and CDDs competence gap	7	Availability of Effective NTDs Service	Service Delivery
48	7	Improve healthcare workers career structure	Inadequate motivation and commitment of healthcare workers and CDDs	7.1	Availability of Effective NTDs Service	Service Delivery
49	7.0	Works closely with village leaders to assign security guards for health posts	Inadequate and insecure drug storage facility at Health Post level	7.4	Facility Infrastructure	Inputs
50	6.8	Establish a conducive drug storage facility at Health posts either by constructing new or renovating existing ones	Inadequate and insecure drug storage facility at Health Post level	7.4	Facility Infrastructure	Inputs
51	6.7	Take immediate corrective action on inappropriate budget users	Poor budget administration, reporting and inappropriate utilization	7.9	Financing	Systems
52	6.7	Delegation of personnel that can effectively lead drug reverse logistics at the district level	Poor drug reverse logistic management	8.2	Drugs & Supplies	Inputs
53	6.7	Performance based incentives	Inadequate motivation and commitment of healthcare workers and CDDs	7.1	Availability of Effective NTDs Service	Service Delivery

54	6.4	Adequate budget allocation for vehicle purchase at districts and PHC facilities	Inadequate means of transportation at district level	6.7	Facility Infrastructure	Inputs
55	6	Early and timely initiation of drug reverse logistic management activities	Poor drug reverse logistic management	8.2	Drugs & Supplies	Inputs
